# Personalised Psychotherapy for Depression: Neuroimaging Predictors of Response to Cognitive Behavioural Therapy

**DOI:** 10.1192/bjo.2026.11102

**Published:** 2026-06-30

**Authors:** Hamed Nagy, Shaima Fadl

**Affiliations:** 1 Bradford District Care NHS Foundation Trust, Bradford, United Kingdom; 2 Tees, Esk and Wear Valleys NHS Foundation Trust, Scarborough, United Kingdom

## Abstract

**Aims:**

Evaluate whether neuroimaging can predict response to cognitive behavioural therapy in those between ages 6–60 years with a diagnosis of major depressive disorder (MDD) and its use in a more personalised treatment plan.

**Methods:**

A literature review was conducted using Ovid database (Medline, Embase, Emcare) and Google Scholar. Relevant studies were identified using a broad search which were then systematically screened. Inclusion criteria were participants aged 6–60 years with MDD, the use of neuroimaging and electrophysiological techniques as biomarkers pretreatment, and assessment of those biomarkers. Eligible literature included randomised controlled trials, cohort, case-control studies, systematic reviews, and meta-analyses published in English. Exclusions were studies with other comorbidities, non-CBT interventions, or combined CBT and pharmacotherapy without separate analyses. Out of 649 identified studies, only nine met the inclusion criteria. The findings were grouped by neuroimaging modality.

**Results:**

Evidence identified fronto-limbic and thalamo-cortical networks as key predictors of CBT response across the nine studies. Task-based fMRI studies showed lower pretreatment activity in subgenual anterior cingulate cortex which predicted superior outcomes. Siegle et al. demonstrated over 75% accuracy in classifying responders and 70% accuracy in remitters (R²=0.29) amongst 49 unmedicated adults. In 22 adults, Richy et al. found greater pretreatment ventromedial prefrontal and anterior temporal activation predicted symptom reduction with post treatment normalisation of fronto-limbic function. Feurer et al. found that greater rostral and subgenual anterior cingulate engagement predicted larger symptom improvement across psychotherapy modalities in 72 adults (B=−0.39, p=0.002).

Dunlop et al. showed subcallosal cingulate connectivity differentiated CBT vs antidepressant responders with 72–78% accuracy for remission and up to 89% accuracy for non-responders in 122 treatment-naive adults. In 30 adolescent participants, Tymofiyeva et al. demonstrated using structural diffusion imaging that greater thalamo-cortical connectivity predicted CBT response with 83% accuracy. Using Neurochemical imaging Dunlop and Mayberg showed that lower anterior insula metabolism predicted CBT response compared to subgenual anterior cingulate hypermetabolism which predicted non-response in 63 adults with MDD. Feurer et al. in 2 separate studies showed that in 112 adolescents with MDD, electrophysiological studies demonstrated that reduced reward positivity, increased late positive potential during reappraisal, and enhanced neural differentiation during self-referential processing predicted CBT outcomes more reliably than self-report measures. A systematic review by Fonseka et al. confirmed consistent involvement of anterior cingulate, prefrontal, insular and limbic regions.

**Conclusion:**

Neuroimaging and electrophysiological biomarkers show promise for predicting CBT response in depression, particularly within anterior cingulate, prefrontal, insular, and thalamo-cortical neural circuits. Despite this, heterogeneity and limitation in replication restrict clinical translation. Larger, prospective, multimodal studies are needed to validate the use of biomarkers in psychotherapy for MDD.

